# Automatic weighing attribute to retrieve similar lung cancer nodules

**DOI:** 10.1186/s12911-016-0313-4

**Published:** 2016-07-21

**Authors:** David Jones Ferreira de Lucena, José Raniery Ferreira Junior, Aydano Pamponet Machado, Marcelo Costa Oliveira

**Affiliations:** Laboratory of Telemedicine and Medical Informatics - HUPAA/UFAL/EBSERH, Computing Institute - Federal University of Alagoas -, Maceio, Brazil

**Keywords:** Content-based image retrieval, Information retrieval, Decision support, Update weighing attributes, Lung cancer

## Abstract

**Background:**

Cancer is a disease characterized as an uncontrolled growth of abnormal cells that invades neighboring tissues and destroys them. Lung cancer is the primary cause of cancer-related deaths in the world, and it diagnosis is a complex task for specialists and it presents some big challenges as medical image interpretation process, pulmonary nodule detection and classification. In order to aid specialists in the early diagnosis of lung cancer, computer assistance must be integrated in the imaging interpretation and pulmonary nodule classification processes. Methods of Content-Based Image Retrieval (CBIR) have been described as one promising technique to computer-aided diagnosis and is expected to aid radiologists on image interpretation with a second opinion. However, CBIR presents some limitations: image feature extraction process and appropriate similarity measure. The efficiency of CBIR systems depends on calculating image features that may be relevant to the case similarity analysis. When specialists classify a nodule, they are supported by information from exams, images, etc. But each information has more or less weight over decision making about nodule malignancy. Thus, finding a way to measure the weight allows improvement of image retrieval process through the assignment of higher weights to that attributes that best characterize the nodules.

**Methods:**

In this context, the aim of this work is to present a method to automatically calculate attribute weights based on local learning to reflect the interpretation on image retrieval process. The process consists of two stages that are performed sequentially and cyclically: Evaluation Stage and Training Stage. At each iteration the weights are adjusted according to retrieved nodules. After some iterations, it is possible reach a set of attribute weights that optimize the recovery of similar nodes.

**Results:**

The results achieved by updated weights were promising because was possible increase precision by 10% to 6% on average to retrieve of benign and malignant nodules, respectively, with recall of 25% compared with tests without weights associated to attributes in similarity metric. The best result, we reaching values over 100% of precision average until thirtieth lung cancer nodule retrieved.

**Conclusions:**

Based on the results, WED applied to the three vectors used attributes (3D TA, 3D MSA and InV), with weights adjusted by the process, always achieved better results than those found with ED. With the weights, the Precision was increased on average by 17.3% compared with using ED.

## Background

**Fig. 1:**
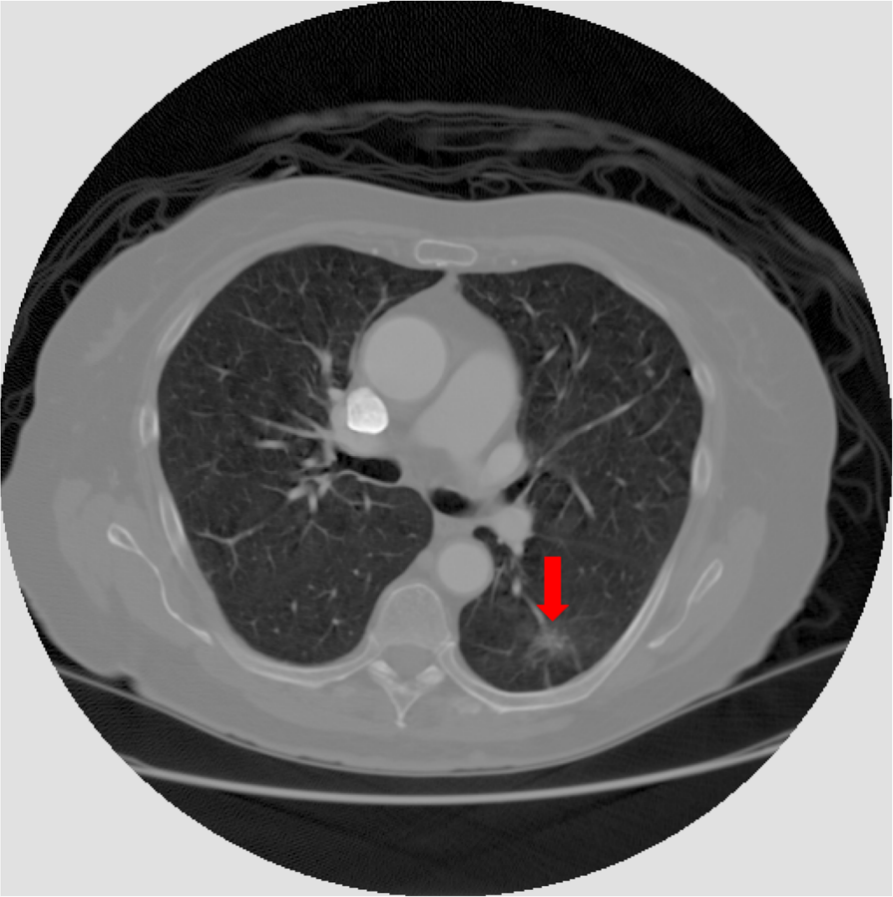
CT image presenting a pulmonary nodule (*red arrow*)

Cancer is a disease characterized as an uncontrolled growth of abnormal cells that invades neighboring tissues and destroys them. Its main manifestation occurs due to the appearance of pulmonary nodules [1]. A nodule is defined as a rounded or irregular opaque figure with a diameter of up to 30 mm, normally presented on a Computed Tomography (CT) image (Fig. 1) [2].

Lung cancer has become the most lethal malignancy in recent decades. Though, despite advances in medicine, there has been little progress regarding the cure of this disease [3]. It is the most common cause of cancer-related deaths, with a 5-year overall survival rate of only 15 % [4]. The main cause is smoking [5]. Thus, the best way to combat this disease is the incentive to smoking cessation and that other people do not become smokers [6].

**Fig. 2:**
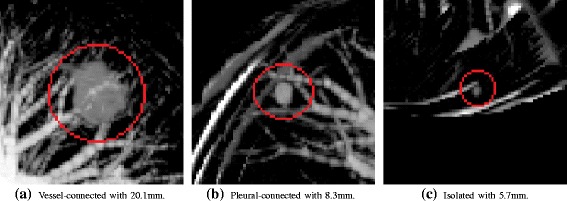
Maximum intensity projection renderings of pulmonary nodules of different sizes [8]. **a**, **b** and **c** are juxtavascular, juxtapleural and isolated nodules, respectively

The diagnosis of lung cancer is a complex task for specialists due to some challenges. One of them is the medical image interpretation process [5]. Inter-observer variation is a recognized challenge and happens due to several aspects, for instance, time constraints, reader’s lack of training and fatigue [7]. Others major challenges of lung cancer diagnosis are the pulmonary nodule detection and classification. Pulmonary nodules can be small, have low contrast in comparison to the lung tissue and be attached to complex lung structures, such as pleural wall of the lung (Fig. 2) [8]. Moreover, classification of benign and malignant nodules depends on their growth rate and change in size from separated CT exams [9]. Therefore, early distinguish of potentially malignant lung nodules is highly important for improving the chance of survival of the patient, without the need to wait for several days.

In order to aid specialists in the early diagnosis of lung cancer, it is necessary to integrate computer assistance to the image interpretation and pulmonary nodule classification processes. The goal of Computer-Aided Diagnosis (CAD) is to improve the accuracy and consistency of image-based diagnosis through computational support used as reference [10]. Techniques of Content-Based Image Retrieval (CBIR) have been described as a promissing CAD tool by helping the specialist in the decision making process as a second opinion. CBIR can provide CAD support by allowing specialists to find previously diagnosed cases from a database that are similar to the cases they are interpreting [11].

However, representation and measurement of similarity from objects in CBIR systems still are considered limitation, because there is not definitive way to do that [7]. Several imaging features were used in the characterization of lung nodules, e.g. texture, shape, size, density, etc. [12–16], but it is still possible to achieve better results because a gold standard has not been found yet.

In the medical domain, texture descriptors become particularly important as they can potentially reflect the fine details contained within an image structure [7]. Moreover, margin sharpness descriptor has been considered important to distinguish nodules in benign and malignant because cancer tumors grow into neighboring tissues [17]. Also different similarity metrics with the same set of attributes may achieve different results in the recovery of similar objects [16].

Ideally, image features should be integrated to provide better discrimination in the comparison process [18]. When specialists classify a nodule, he is supported by information from exams, images, electronic medical records and others [19]. But each information has more or less weight over decision making about nodule malignancy. In this context, there are attributes with more or less influence in nodule classification, which introduces a semantic factor to image retrieval problem. Thus, finding a way to measure the weight allows improvement of image retrieval process through the assignment of bigger weights to that attributes that best characterize the nodules.

The aim of this work is to present a method to automatically calculate attribute weights based on local learning to reflect the interpretation on similar pulmonary nodule retrieval process. Moreover, to evaluate the accuracy of the algorithm, pulmonary nodules were represented through vectors of 3D Texture Attributes (3D TA) and 3D Margin Sharpness Attributes (3D MSA). The analysis of these attributes will enable identify which of these vectors provide a better accuracy in the recovery of similar nodes. Finally, we want confirm the hypothesis that the Weighted Euclidean Distance with adjusted weights provides better results than Euclidean Distances.

The remainder of this paper is organized as follows: “Related works” section presents a brief overview of literature of algorithms applied to CBIR systems using the concept associated to attributes; “Global and local weighing” section summarizes the main concepts related to updating weights that are necessary to facilitate the comprehension of proposed method; “Methods” section describes the algorithm proposed as well the database, attributes extracted and similarity metric used; “Results and discussion” section details the results achieved and compare it with others from literature associated; finally, “Conclusion” section presents conclusion of this work and limitations of the algorithm.

## Related works

In the current literature, according to our knowledge, few studies address the measure of weights and different similarity metrics applied to the context of CBIR. Among them, the work presented by [12] describes an algorithm that extracts attributes from the information of margin sharpness of the nodules. These authors represent nodules through vectors constructed from histograms of the window and scale attributes. The calculation of these attributes starts from lines drawn on the margin of all slices of nodules of CT scans, they are drawn in control points on the lesions margin. After extraction of these lines, [12] record the values of pixels intensity values in line segments. Then, they apply a Sigmoid Function to fit the values using a weighted nonlinear regression function. From this function were calculated two values, which are used to characterize each line segment: window and scale.

DHARA et al. [14] presents a proposal for CBIR system to recover solid pulmonary nodules with size between 3 and 30 mm. To represent nodules were used shape (roundness, lobulation index, speculation index, mean radial distance, calcification index and 3D accutance nodule surface) and texture (contrast, entropy and cluster trends, homogeneity and texture classification of the internal tissue) attributes from the reconstructed 3D nodules. They also used other nine characteristics associated with nodules (texture, subtlety, speculation, lobulation, sphericity border malignancy, internal structure and calcification). According to these authors, only some features are useful to represent the images. So they used logistic regression to find the subset of attributes that allow higher discrimination using the criterion of maximum relevance and minimum redundancy, however, the authors did not indicate the attributes that provided higher discrimination. Finally, the similarity metric used to retrieve and ranking nodules was Euclidean Distance (ED).

SEITZ et al. [15] describes a CBIR system in combination with genetic algorithms to determine the optimal combination of image attributes to increase the accuracy in retrieval of similar nodules. Sixty three attributes were extracted from texture (using Gabor filter, Markov Random Fields and attributes proposed by Haralick from Coocurrence Matrix (COM)), size, shape and intensity to represent vectorially nodules. The similarity metric used was the ED.

KURUVILLA & GUNAVATHI [16] present other work where was used a CBIR system to retrieve exams with similar pulmonary nodules in order to find the best set of attributes that describe the nodules, according to the parameters used to calculate the accuracy of the neural network algorithm. Moreover, the authors evaluated different similarity metrics to identify the higher accuracy in recovering nodules. Two sets of attributes were calculated: attributes of COM and statistical attributes. The attributes calculated from COM were energy, entropy, dissimilarity, contrast, inverse difference, correlation, homogeneity, autocorrelation, cluster shadow, prominence cluster, maximum probability, sum of squares, sum average, sum variance, sum entropy, variance difference, the entropy difference, correlation measure of information, maximum correlation coefficient, standard inverse difference and normalized reverse difference moment. The statistical attributes were the mean, standard deviation, skewness and kurtosis. Among all calculated attributes, were selected as most relevant by Neural Network: autocorrelation, contrast, correlation, cluster shadow, prominence cluster, dissimilarity, energy, entropy, homogeneity, sum variance and asymmetry. The metrics used were ED, Manhattan Distance, City Block Distance, Chebychev Distance, Tversky Distance, Canberra Distance, Bray-Curtis, Chi Squared Distance and Squared Chord Distance.

Finally, HAN et al. [20] presented a predictive model built using Support Vector Machine (SVM) with a Radial Basis Function kernel (RBF). The model evaluation was performed by calculating the sensitivity and specificity and presented by the Area Under Curve (AUC). The attributes used by them were classified into three types: Texture Attributes; Gabor Attributes; Local Binaries Patterns. Texture Attributes were calculated from the 2D and 3D COM. It is noteworthy that this analysis did not take into account a CBIR system, but a classification model.

## Global and local weighing

In most learning methods, a single global model is used to fit all the training base, while local models attempt to fit the training base only in the region around the reference point of the search. Some examples of local learning algorithms are K-Nearest Neighbor (KNN), Weighted Average (WA) and Locally Weighted Regression (LWR). Each of these models combine near objects from reference object to estimate the appropriate output. KNN models use neighborhood objects to reference object to determine the output value. WA assigns weights to objects close to the reference object that are inversely proportional to the distance between them in the space n-dimensional. LWR set the near object by means of a regression weighted distance [21].

Our proposal has characteristics indicated by ATKESON et al. [21] which refer to Locally Weighted Learning (LWL). Concisely, LWL is a concept that refers to lazy learning systems with the aim of building models resulting from approximation function through weight adjustment in polynomial functions. The purpose of LWL is emphasizing data that is similar to the reference object, and de-emphasize data that are dissimilar, rather than treat all data equally. The requirements cited by [21] for a system to be LWL type are: distance function, LWL systems require a relevant measure, which can be measured using a distance measurement; separate criteria, LWL systems calculate weights from each training object; classified objects, each object must have associated with it an appropriate output, which for the output class models should be a label and for regression models, the output should be an expected value; representation, each object is represented by a vector of fixed size values (symbolic or numeric) for a specific list of features.

## Methods

### Pulmonary nodule image database

The pulmonary nodule image database used in this work is available in [22]. This nodule database has CT scans provided by the Lung Image Database Consortium and Image Database Resource Initiative (LIDC-IDRI). LIDC-IDRI is publicly available database for the medical imaging informatics community and consists of cases with marked-up lesions with annotations, including nodules outlines and subjective lesion feature ratings [23]. It has associated specialists annotations, including nodule outlines and subjective nodule characteristic ratings.

**Fig. 3:**
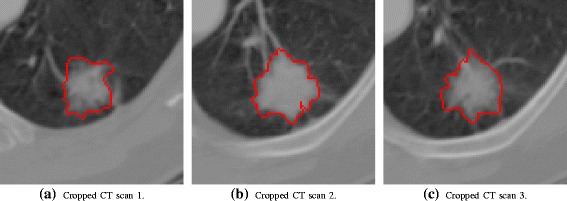
Pulmonary nodule sample of a 3-slice volume, with LIDC-IDRI radiologist’s marks

LIDC-IDRI project required 4 experienced specialists to review each image of a CT series with a graphical user interface and outlines lesions that they considered to be a nodule in the range of 3 to 30 mm. However, for the purposes of this work and the development of the public database, only the reading of the specialist that identified the highest number of nodules was inserted in the database. Each nodule outline was meant to be a localizing “outer border” so that, in the opinion of the specialist, the outline itself did not overlap pixels belonging to the nodule (Fig. 3) [23].

Each specialist defined an integer value on a 1 to 5 scale for the lesion’s likelihood or probability of malignancy, in which 1 is highly unlikely for cancer, 2 is moderately unlikely for cancer, 3 is indeterminate likelihood, 4 is moderately suspicious for cancer and 5 is highly suspicious for cancer.

The image database used has 752 exams and 1,944 lung nodules. Among the nodules, those with malignancy 3 were discarded because they are associated with an unspecified malignant. Thus, the initial value 1,944 goes to 1,171 when chosen nodules with malignancy 1, 2, 4 and 5 (Table 1).

**Table 1:** Number of nodules associated to each malignancy

Malignancy probability	1	2	4	5	Total
Number of nodules	273	472	266	160	1.171

We divided the database in three parts: 1 database for training purposes, composed of 65 nodules of each malignancy resulting in 260 nodules; 1 database for evaluation purposes, composed of 65 nodules of each malignancy resulting in 260 nodules; and 1 database validation purposes, composed of 30 nodules of each malignancy resulting in 120 nodules. The determination of the size of the bases mentioned above is related to smaller number of nodules among malignancies used, ie, was taken as a reference the amount of malignancy 5 that has 160 nodules (Table 1) and this value was divided into three pieces as explained. As other malignancies have a larger number of nodes, from these we randomly selected the same quantities of nodules.

The first two databases were used during training and evaluation of weights. While the third database (validation) was used to validate the best weights found. The last database is important to ensure that the best weights found were not only for the evaluation database, which it would produce overfitting.

### Pulmonary nodule feature extraction

We extracted 48 attributes from the 1.171 nodules. They are distributed in 12 3D MSA and 36 3D TA, which will be explained below. 3D AT were extracted from manually segmented margin of nodules by specialists involved in LIDC-IDRI project as well as 3D MSA. The region of interest in exams is the region marked at the margin of nodules. Thus, the original size of the nodules contained in the exams were used.

**Fig. 4:**
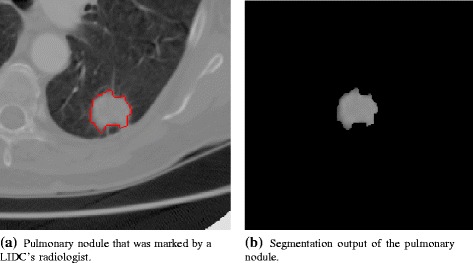
Manual segmentation of a pulmonary nodule from the LIDC-IDRI [22]

**Fig. 5:**
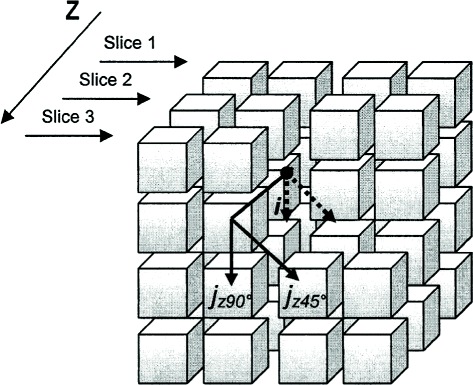
GLCM calculation over a 3-slice image volume [24]. Between-slices joint relationships have 1 pixel and slice distances in 45° and 90°

In order to extract textural features, a 3D texture analysis was applied to manually segmented pulmonary nodules. All nodules had lesion images segmented using the radiologist’s marks (Fig. 4). After the segmentation, texture attributes were extracted from the voxels using the gray level COM. COM is a technique to extract information from second-order statistical texture. The COM method obtains from a single image the occurrence probability of a pixel pair with intensity *i,j* and spacing between the pixels of *Δx* and *Δy* in the dimensions *x* and *y*, respectively, given a distance *d* and orientation *θ* [11]. Calculation of the COM in a volume of images extends the evaluation of the probability function to the rectangular Z-axis, in order to study between-slices joint probabilities on an image volume composed of multiple slices (Fig. 5) [24]. Second-order histogram statistics are applied to the COM producing the texture attributes. TA used in this work were suggested by Haralick et al. [25], and are listed below:

1 $$ \text{Energy} = \sum\limits_{i,j} C^{2}(i,j),  $$

2 $$ \text{Entropy} = - \sum\limits_{i,j} C(i,j) logC(i,j),  $$

3 $$ \text{Inverse difference moment} = \sum\limits_{i,j} \frac{C(i,j)}{1 + (i - j)^{2}},  $$

4 $$ \text{Inertia} = \sum\limits_{i,j} \left(i - j\right)^{2} C(i,j),  $$

5 $$ \text{Variance} = \sum\limits_{i,j} \left(i - \mu\right)^{2} C(i,j),  $$

6 $$ \text{Shade} = \sum\limits_{i,j} \left(i + j - \mu_{x} - \mu_{y}\right)^{3}C(i,j),  $$

7 $$ \text{Promenance} = \sum\limits_{i,j} (i + j - \mu_{x} - \mu_{y})^{4}C(i,j),  $$

8 $$ \text{Correlation} = - \sum\limits_{i,j} \frac{(i - \mu_{x})(j-\mu_{y})}{\sqrt{\sigma_{x}\sigma_{y}}}C(i,j),  $$

9 $$ \text{Homogeneity} = \sum\limits_{i,j} \frac{C(i,j)}{(1 + \mid i - j\mid)},  $$

where *C*(*i,j*) are the elements from the COM, *μ*_*x*_ and *μ*_*y*_ are the mean, *σ*_*x*_ and *σ*_*y*_ are the standard deviation, obtained by the following equations:

10 $$ \mu_{x} = \sum\limits_{i}iC_{x}(i),  $$

11 $$ \mu_{y} = \sum\limits_{j}jC_{y}(j),  $$

12 $$ \sigma_{x} = \sum\limits_{i}(i - \mu_{x})^{2} \cdot \sum\limits_{j}C(i,j),  $$

13 $$ \sigma_{y} = \sum\limits_{j}(j - \mu_{y})^{2} \cdot \sum\limits_{i}C(i,j),  $$

14 $$ C_{x}(i) = \sum\limits_{j}C(i,j),  $$

15 $$ C_{y}(j) = \sum\limits_{i}C(i,j).  $$

Thus, a texture feature vector can be obtained by means of the calculation of the nine attributes (Eqs. 1–9) applied to the COM in orientations 0°, 45°, 90° and 135°, for instance. In this case, each nodule can be associated with a 36 dimensions texture feature vector.

**Fig. 6:**
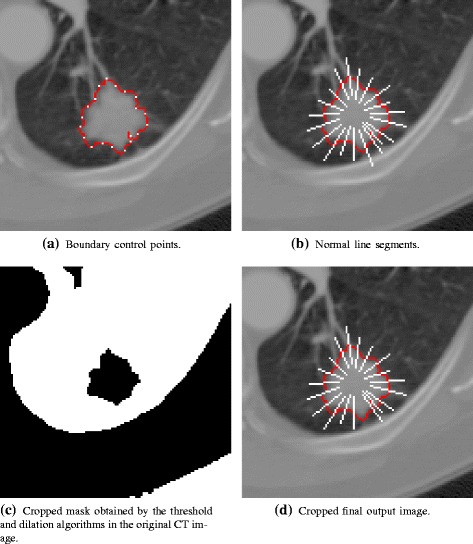
Output images from the 3D margin sharpness analysis

A 3D margin sharpness analysis was implemented to characterize the lung nodules and proposed in [12], in which a data statistical analysis was performed by extracting features from a sorted array composed of the gray level values of the pixels belonging to perpendicular lines drew over the edges on all nodule slices. The implementation is as follows: twenty control points were automatically selected on the marked lesion edge, starting by the first point marked by the specialist (Fig. 6a). If the boundary has *p* pixels, than a control point is marked every $\frac {p}{20}$ pixels. The authors have not demonstrated the reason that they was used this amount of control points. Normal lines were drawn at each of the 20 control points across the nodule boundary (Fig. 6b). A mask was created to eliminate the line segments that cross the lung wall because, otherwise, it will introduce pixel information that does not belong to the nodule or lung tissues. The mask was generated by applying a threshold algorithm along with morphological dilation operation in the original CT image (Fig. 6c). After excluding normal line segments that do not belong to the lung by means of the lung mask application (Fig. 6d), pixel intensities from the remaining line segments from all nodule images were recorded in a single sorted array. Then a data statistical analysis was performed by extracting statistical attributes from the pixel intensities sorted array. The margin sharpness feature vector was composed by the attributes listed in Eqs. 16–27, in which *a* is the pixel intensities array of size *n*, *a*_1_ is the intensity value of a pixel outside the nodule and *a*_*n*_ is the intensity value of a pixel inside the nodule. Therefore, each nodule is characterized as a 12-dimension margin sharpness feature vector.

16 $$\begin{array}{@{}rcl@{}} \text{Difference of two ends} &=& a_{n} - a_{1}, \end{array} $$

17 $$\begin{array}{@{}rcl@{}}  \text{Sum of values} &=& \sum\limits^{n}_{i=1}a_{i}, \end{array} $$

18 $$\begin{array}{@{}rcl@{}}  \text{Sum of squares} &=& \sum\limits^{n}_{i=1}{a_{i}^{2}}, \end{array} $$

19 $$\begin{array}{@{}rcl@{}}  \text{Sum of logs} &=& \sum\limits^{n}_{i=1}\log a_{i}, \end{array} $$

20 $$\begin{array}{@{}rcl@{}} \text{Arithmetic mean} (\mu) &=& \frac{1}{n}\sum\limits^{n}_{i=1}a_{i}, \end{array} $$

21 $$\begin{array}{@{}rcl@{}} \text{Geometric mean} &=& \sqrt[n]{\prod\limits^{n}_{i=1}a_{i}}, \end{array} $$

22 $$\begin{array}{@{}rcl@{}} \text{Population variance} &=& \frac{1}{n}\sum\limits^{n}_{i=1}\left(a_{i} - \mu\right)^{2},  \end{array} $$

23 $$\begin{array}{@{}rcl@{}} \text{Sample variance ({v})} &=& \frac{1}{n - 1}\sum\limits^{n}_{i=1}(a_{i} - \mu)^{2},  \end{array} $$

24 $$\begin{array}{@{}rcl@{}} \text{Standard deviation ({s})} &=& \sqrt{v},  \end{array} $$

25 $$\begin{array}{@{}rcl@{}} \text{Kurtosis measure} &=& \frac{\frac{1}{n}\sum\limits_{i=1}^{n} \left(a_{i} - \mu\right)^{4}}{s^{4}},  \end{array} $$

26 $$\begin{array}{@{}rcl@{}} \text{Skewness measure} &=& \frac{\frac{1}{n}\sum\limits_{i=1}^{n} (a_{i} - \mu)^{3}}{s^{3}}, \end{array} $$

27 $$\begin{array}{@{}rcl@{}} \text{Second central moment} &=& \frac{\frac{1}{n}\sum\limits_{i=1}^{n} (a_{i} - \mu)^{2}}{s^{2}}. \end{array} $$

### Attribute normalization

Each extracted attribute has its own range of values (scale) and are not necessarily the same. In order to use distance-based similarity metrics, it is necessary to normalize the data to put it all in a specific scale because that metrics are sensible to different scales [26].

SHALABI et al. [27] point that there are many methods for data normalization including: Min-Max Normalization, which performs a linear transformation on the original data; Z-score Normalization, which normalizes the initial values based on the mean and standard deviation (SD) of the sample; and Normalization by Decimal Scale, which normalizes changing the scale by moving the decimal point of the sample values.

In this work we use Z-score normalization method (Eq. 28). This is one of the most widely used methods in the literature for normalization values. Its range of values is defined in [−3,+3], and the values are determined by the SD and the average of the sample values. The Z-score value identifies where a particular value is (up or down) with respect to average in the normal distribution curve from SD [26, 28].

28 $$ Z = \frac{X - \overline{x}}{\sigma},  $$

where *Z* is the vector with normal distribution with; *X* is the vector with original values from attributes; $\overline {x}$ is the mean of attribute values; *σ* is the deviation pattern.

### Similarity distance metric

One of the biggest challenges for CBIR systems is how to properly define the assessment of similarity used to index the database and/or make the ranking based on the similarity of retrieved images according to a given search criteria [7]. This is because the accuracy in image retrieval is strongly influenced not only by the attributes chosen to represent the objects, but also by the similarity measure used [29]. What leads the need to define the distance function that allows retrieve the most similar images according to the domain of search space [30].

A common method is to employ vector distance in multidimensional space, usually an Euclidean Space, in which an image is represented by vectors of descriptors/attributes [7]. In this context, basically all systems use the assumption that there is equivalence between the image and the attributes vector. These systems often use metrics which are easily understandable for measuring the distance between the reference image and the possible similar images that result of the search. All of it represented by feature vectors in a *n*-dimensional space [31].

A distance function *d*() evaluates the distance, or dissimilarity, between a pair of elements and it should meet the following properties [29, 30], where $\mathbb {V}$ is a *n*-dimensional vector defined in Euclidean space:

1. Symmetry: $\{ \forall v \in \mathbb {V} | d(v_{1}, v_{2}) = d(v_{2}, v_{1})\}$;
2. Non-negativity: $\{ \forall v \in \mathbb {V} | 0 \leq d(v_{1}, v_{2}) < \infty \}$;
3. Triangular inequality: $\{ \forall v \in \mathbb {V} | d(v_{1}, v_{2}) \leq d(v_{1}, v_{3}) + d(v_{3}, v_{2})\}$.

Intuitively, the smaller distances correspond to higher similarity. Thus, as closer to zero the distance value is, higher is the similarity of objects according to the criteria used by the image descriptors. Inversely, the higher is the distance value, smaller is the similarity [32].

The similarity metric used in this work is WED, presented in Eq. 29. WED is a variation of ED that has weights associated to vector coordinates. This function is used to measure the similarity of a nodule pair through the comparison of their attributes vectors. Nodules with higher similarity degree have the smallest values obtained by the similarity distance measure.

29 $$ d(\stackrel{\rightarrow}{x},~\stackrel{\rightarrow}{y}) = \sqrt{w_{1}(x_{1} - y_{1})^{2} + \dots + w_{a}(x_{a} - y_{a})^{2}},  $$

where $\stackrel {\rightarrow }{x} = \,[x_{1}, \dots, x_{a}]$ is the attributes vector from reference image, $\stackrel {\rightarrow }{y} =\, [y_{1}, \dots, y_{a}]$ is the attributes vector from images that will be compared with reference image, $\stackrel {\rightarrow }{w} = [w_{1}, \dots, w_{a}]$ is the weights vector associated to each of attributes and *a* is the number of attributes used (Eq. 29).

Weights represent the influence from attributes in similar nodules retrieval process by CBIR. It leads to the following induction: biggest values are associated to attributes that have homogeneous values, and lower values are associated to attributes whose values are very heterogeneous. If Energy attribute, for instance, has low variability in values of retrieved nodules from a specific malignancy to it will be assigned bigger weight than another that has high variability. This way is possible retrieve nodules with attributes values increasingly similar to the detriment that are not.

Identifying which attributes carry the most relevant information to lesion classification allows the achievement of better results in accuracy of medical diagnosis by providing more accurate results in image retrieval algorithms.

### Automatic weighing updating process

**Fig. 7:**
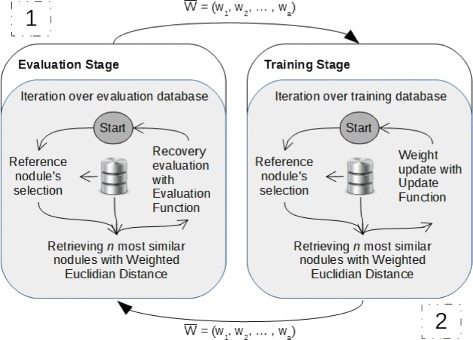
Workflow of the weighing update process

The process consists of two stages that are performed sequentially and cyclically: an Evaluation Stage and a Training Stage (Fig. 7).

The stages has similar structures that consist basically in apply Leave-One-Out (LOO) [33] to iterate over correspondent databases selecting each stored nodule and using it as a reference nodule to retrieve *n* most similar cases through of a similarity function (“Similarity distance metric” section).

Cycle begins with Evaluation Stage (process 1 of Fig. 7) having as initial weights a set *W*= [*w*_1_,…,*w*_*a*_], where $\{w \in R_{+}^{*} | w = \frac {1}{a}\}$ and *a*∈*N*^∗^ corresponds to the number of attributes used in nodule representation. At the end, we have evaluation value *v*_1_ from this set (*W*) measured from an evaluation function.

Then, Training Stage (process 2 of Fig. 7) starts receiving as initial weights the values used in the past Evaluation Stage and, thenceforward, start the weighing update process. At the end of this stage, a new set of weights (*WCurrent*) is determined and will be passed to the Evaluation Stage to determine its evaluation value *v*_2_. The comparison between *v*_1_ and *v*_2_ identify whether the weight changes increased or decreased the similar nodule retrieval precision.

This cycle must be performed until any stopping criteria is reached. During the cycles, the set *W* with weights that reach best evaluation value is stored and, at the end, is pointed as that best adjust the similarity metrics used. The adopted criterion was that the process stops after 100 training iterations and evaluation without there were improvements in the evaluation results. That is, after obtaining a maximum value of the function evaluation after *i* iterations, if this value is not increased in 100 subsequent iterations, the weighing update process stops and indicate the highest rating as the ideal weights for the attribute vector.

### Evaluation stage

This stage consists in iterating over evaluation databases by LOO using each nodule as a reference nodule to retrieve the *n* most similar. At each retrieval the evaluation value *v* is calculated from the Eq. 30, which is an exponential decay function, where:

- *R*_*n*×*a*_ is an ordered matrix with nodules retrieved where *n* is the number of similar nodules retrieved and *a* is the number of used attributes. The order of matrix is determined by the nodule similarity, the most similar stay at initial position;
- *s*_*i*_ is the reward value associated to relevance of nodule *n* from matrix *R* at position *i*;
- {*γ*∈*R* | 0<*γ*≤1} is the discount factor that adjusts the reward relevance *s* given over retrieval ranking.
  30 $$ f (R_{n \times a}) = \sum\limits_{i=1}^{n}{\gamma^{i} s_{i}}.  $$

This evaluation function was adopted due to its ability to represent the amortization of reward over the retrieving order. This is an important property to our proposition because with the large amount of retrieved exams the users tend to evaluate the best placed results and it will guide the specialist in diagnostic [31, 34].

Reward applied to the nodules depends of reference and retrieved nodule malignancy. The values assigned are following: 4, if it is highly relevant; 2, if it is moderately relevant; and 0, if it is highly or moderately irrelevant. A reward policy was defined to privilege the relevant nodules, and not rewarding those who do not meet this condition. Relevance is determined as follows: if the reference nodule have malignancy 5 or 4, malignancies of recovered nodules will be highly relevant if they malignancy 5, moderately relevant if they malignancy 4, moderately irrelevant if they malignancy 2 and highly irrelevant if they 1 malignancy; if the reference node malignancy have 1 or 2, malignancies of recovered nodules will be highly relevant if they malignancy 1, moderately relevant if they malignancy 2, moderately irrelevant if they malignancy 4 and highly irrelevant if they malignancy 5. To nodules highly and moderately irrelevant are assigned the reward value of 0 (zero), because these can induce the specialist to error. Then, they were not given positive reward.

### Training stage

Training Stage aims to find a weight set *W* associated to the attributes that allow a nodule retrieval in which the cases are more similar as possible. Nodules are considered to be similar when the values of each attribute are close or even the same.

The weights associated to the attributes reflect the different contribution of descriptors in nodule characterization. There is not a direct mapping between the classification criteria used by specialists and the nodule representation used by computer. Our aim with this work is to find a weight adequation such that it is possible reach better results in similar nodule retrieval through minimizing the influence of the attributes that has high dispersion rate in nodules with same malignancy and maximizing the influence of the attributes that has low dispersion rate in nodules with same malignancy.

The proposed update weighing is based on the Standard Deviation (SD) (*σ*) to adjust the weights. The SD is a measure of statistical dispersion, that is, it measures the scattering of data from a sample with respect to its average. The idea is based in following assumptions: if all similar nodules have similar values for a given attributes vector, it means that these are good indicators to represent these nodules. On the other hand, if the values of a attributes vector are very different, that is widely dispersed, so they are not good indicators. Therefore, the Inverse Standard Deviation (ISD) (Eq. 31) of data associated with an attribute can be considered a good estimate of it weight because the smaller the variance, the higher is the weight and vice versa.

31 $$ w(a) = \sigma^{-1}.  $$

**Fig. 8:**
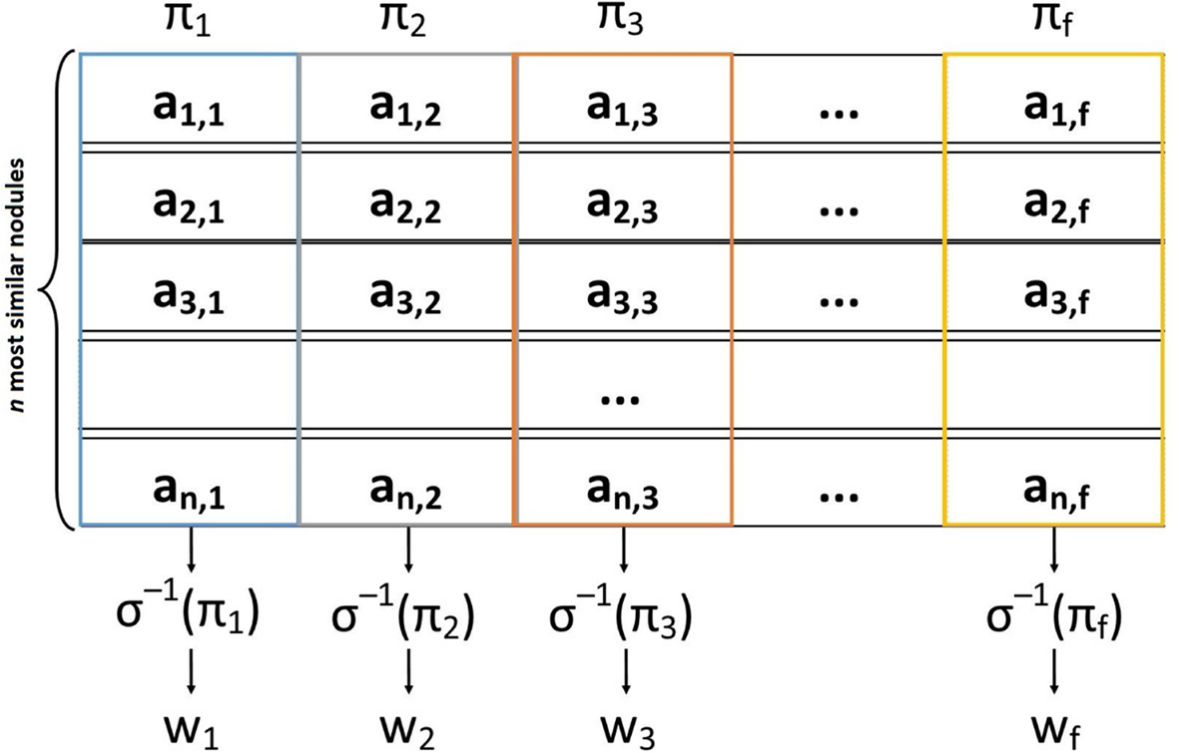
Update weighting methodology for the *n* retrieved nodules. *f* is the number of attributes used to represent the nodules, *Π*_*f*_ represents the projection of attribute *f* over nodules retrieved matrix, *σ* ^−1^(*Π*_*f*_) represents the application of inverse of deviation pattern over the resulting sample from projection *Π*_*f*_, and *w*_*f*_ is the weight of attribute *a*_*f*_

The updating process is performed based on *n* retrieved nodules at each iteration over training database. From the nodule matrix *R*_*n*×*a*_ retrieved during training, projections are made over each columns correspondent to the nodule attributes and calculated the weights through this projections with *σ*^−1^ (Fig. 8).

After the identification of each *w*_*f*_ associated to the *a*_*f*_ attributes, it is necessary to apply the Eq. 32, because $w_{f} \in R^{*}_{+}$ and it can assume values very large when the sample has low variation, or tiny values when the sample has large variation. Thus, we have a new weight *w*^′^*f* with range (0,1].

32 $$ w'_{f} = \frac{w_{f}}{\sum_{i=1}^{f} w_{i}}.  $$

The vector of weight resultant from each recovery is called current weight (*WCurrent*). For the process take into account the various iterations over the database, it is necessary the existence of a weight set that are adjusted iteratively. At each iteration we find a *WCurrent*, which is used to adjust the set *W* with the Update Function (Eq. 33):

33 $$ W^{*} = W + \alpha (W - WCurrent),  $$

where *W* is the set with the best weights until that moment, *WCurrent* is the set with the weights from current iteration, *α* is the adjust factor and *W*^∗^ is the new set of weights.

The lazy learning characteristic in this proposal is related to the adjustment rate of weights (*α*), which determines how much will be learned in each iteration in the Training Stage. As can be seen in Eq. 33, the weight vector *WCurrent* determined in LOO iteration over the training base is the result of the calculation of the weights in current iteration, while the weight vector *W* is the vector calculated from all previous iterations. *α* applied in the difference between *W* and *WCurrent* implies how much the difference between learning memory (*W*) and learning from current iteration (*WCurrent*) will influence the final learning memory (*W*^∗^).

And LWL characteristic in this work refers to the number of recovered nodules (*n*) during the Training Stage that were used to calculate the weight of attributes through the ISD. Because of this feature, only the closest nodules of the reference nodule are used to adjust the weights of the attributes in order to emphasize nodules of the same class, and deemphasizing nodules with different classes, by assigning larger weights to those attributes which provide recovery nodules attribute values with lower dispersion index. Note that the emphasizing of similar nodules is achieved indirectly based on the premise that the same malignant nodules are similar and, therefore, it have attributes vectors also similar (low variability), that is, is not used a criteria based on the class to determine the weights of the objects as in KNN and WA models.

LWL is critically dependent of the distance function used and the function does not necessarily need to satisfy formal mathematical requirements for distance metrics. The variation of the ED with the introduction of the weights associated to dimensions of the objects is to influence the values. Assign zero value for a dimension is the same as ignore it on the distance function. Because of this, it is adopted WED. The maximum value from weighted function should be achieved with zero distance, and this value grown up smoothly when the distance increases. Moreover, weighting functions should always be non-negative, because negative values can lead to a higher error rate during training. However, the final weights can be positive or negative [21].

## Results and discussion

Aimming to analyze the results we used the Precision vs. Recall (PR) and Precision(*n*) (PN) methods. They are calculated from all nodules retrieved from Evaluation and Validation database through Leave-One-Out (LOO). [35] define Precision (Eq. 34) as the percentage of recovered objects that are relevant, and Recall (Eq. 35) as the percentage of relevant objects recovered. Relevant object is one that is of interest in a given context.

34 $$\begin{array}{@{}rcl@{}} \text{Precision} &=& \frac{\text{Number of relevant objects retrieved}}{\text{Total number of objects retrieved}} \end{array} $$

35 $$\begin{array}{@{}rcl@{}} \text{Recall}&=& \frac{\text{Number of relevant objects retrieved}}{\text{Total number of relevant objects}} \end{array} $$

Three different feature vectors were used in order to evaluate our proposal (“Pulmonary nodule feature extraction” section). The first vector has 36 3D extracted through 3D COM. TA were extracted because they are a traditional technique in CBIR systems. The second vector has 12 3D MSA extracted from the margin sharpness analysis. 3D MSA were extracted due to its potential to characterize pulmonary nodules according to potential malignancy. And the third vector has 48 Integrated Vector (InV), which are composed of concatenated 3D TA and 3D MSA.

In order to evaluate the accuracy with defined methods (PR and PN), malignancies associated with nodules were grouped as benign or malignant. Thus, nodules with probability of malignancy 1 and 2 were grouped as benign and malignant nodules with probability 4 and 5 were grouped as malignant. It is noteworthy that the nodules with malignancy 3 were discarded because it have not classification defined.

To demonstrate the capabilities of the algorithm proposed here and prove the accuracy of the results achieved were defined some configurations to test varying the main parameters of the algorithm, which is the adjustment rate, the discount factor and the number of nodules recovered applied to combinations of 3 attribute vectors that are 3D TA, 3D MSA, and the concatenation of these 2 vectors resulting in InV extracted from nodules. These tests resulted in graphics (PR and PN) that were the basis for our discussion. Then, due to the large number of combinations involving the parameters of algorithm and the vector attributes, given that $\{n \in \mathbb {N}~|~0~<n \leq \text {number of nodules in the database}\}$ and $\{ \alpha,~\gamma \in \mathbb {R}~|~0 <~\alpha, \gamma < 1 \}$ was defined as reference values for *n*, *α* and *γ* respectively 15, 0.3 and 0.8, and two others sets of values being one greater and other smaller than the reference value. These values were set to reduce the number of possible combinations of the attributes vectors and parameters of the algorithm, but still enabled the analysis for precision measurement. The determination of the reference values was empirically, because it provided better results as precision as well execution time of algorithm for the various tests performed during development. Table 2 summarizes the configurations used in our tests.

**Table 2:** Summary of configurations defined for the tests, where *α* is the adjustment rate, *γ* the discount factor and *n* the number of retrieved nodules

TA			WSA			InV		
*n*	*α*	*γ*	*n*	*α*	*γ*	*n*	*α*	*γ*
10	0.2	0.7	10	0.2	0.7	10	0.2	0.7
**15**	**0.3**	**0.8**	**15**	**0.3**	**0.8**	**15**	**0.3**	**0.8**
20	0.4	0.9	20	0.4	0.9	20	0.4	0.9

Bolded values refer to values that, empirically, reached good values during development and test of the algorithm. So, we defined it as the reference to generate the test configuration

PR results using 3D TA vector with the initial weights using only the Evaluation database achieve Precision of 87 % with Recall 25 %, Precision of 64 % with Recall 50 % and Precision of 50 % with Recall 75 % when retrieve benign nodules. When retrieve malignant nodules was obtained Precision of 91 % with Recall 25 %, Precision of 86 % with Recall 50 % and Precision of 74 % with Recall 75 %. The PN achieve Precision of 87 % for benign nodules and 93 % for malignant nodules for retrive until the thirtieth nodule. After update weights, Precision was 98 % with Recall 25 %, Precision of 97 % with Recall 50 % and Precision of 97 % with Recall 75 % when retrieve benign nodules, and when retrieve malignant nodules was obtained a Precision of 99 % with recall 25 %, Precision of 99 % with Recall 50 % and Precision of 96 % with Recall 75 % in the Evaluation database. While with Validation database, the Precision was 98 % to the Recall 25 %, Precision 98 % to the Recall 50 % and a Precision of 97 % to the recall 75 % when retrieve benign nodules, when retrieve malignant nodules, was obtained an Precision of 100 % to the Recall 25 %, Precision 100 % to the Recall 50 % and Precision of 97 % to the Recall 75 %. The PN also was improved, achieving Precision of 98 % in the recovery of benign nodules and malignant using the two databases (Evaluation and Validation) to retrieve 30 nodes at each database.

Tables 3 and 4 present a summary of the evaluation results in the Validation database using PR and PN from 3D TA vector with and without update weights respectively.

**Table 3:** Summary of results using PR and PN obtained with the 3D TA vector without update weights applied to Validation database

	Recall		
	25 %	50 %	75 %
Precision to benign	87 %	64 %	50 %
Precision to malignant	91 %	86 %	74 %
	Precision (*n*=30)		
Benign	87 %		
Malignant	93 %

**Table 4:** Summary of results using PR and PN obtained with the 3D TA vector with update weights applied to Validation database

	Recall		
	25 %	50 %	75 %
Precision to benign	98 %	97 %	97 %
Precision to malignant	99 %	99 %	96 %
	Precision (*n*=30)		
Benign	98 %		
Malignant	98 %		

3D MSA vector with the initial weights using the Validation database achieve Precision of 96 % with Recall 25 %, Precision of 86 % with Recall 50 % and Precision of 64 % with recall 75 % when retrieve benign nodules. When retrieve malignant nodules, achieve Precision of 96 % to the Recall 25 %, Precision of 83 % to the Recall 50 % and Precision of 60 % to the Recall 75 %. PN achieve Precision of 96 % when retrieve benign nodules and Precision of 95 % when retrieve malignant nodules for retrieval of nodes 30 each malignancy. After update weights achieve Precision of 100 % to the Recall 98 %, for both for the retrieve of benign nodules and for malignant nodules in the Evaluation and Validation databases, and PN was maintained with Precision of 100 % to retrieve 30 nodules, benign and malignant, in the Evaluation and Validation databases.

Tables 5 and 6 present a summary of the evaluation results in the Validation database using PR and PN from 3D MSA vector with and without update weights respectively.

**Table 5:** Summary of results using PR and PN obtained with the 3D MSA vector without update weights applied to Validation database

	Recall		
	25 %	50 %	75 %
Precision to benign	96 %	86 %	64 %
Precision to malignant	96 %	83 %	60 %
	Precision (*n*=30)		
Benign	96 %		
Malignant	95 %

**Table 6:** Summary of results using PR and PN obtained with the 3D MSA vector with update weights applied to Validation database

	Recall		
	25 %	50 %	75 %
Precision to benign	100 %	100 %	100 %
Precision to malignant	100 %	100 %	100 %
	Precision (*n*=30)		
Benign	100 %		
Malignant	100 %		

Finally, the InV vector with the initial weights using Evaluation database achieved Precision of 82 % to the Recall 25 %, Precision of 66 % to the Recall 50 % and a Precision of 52 % to the Recall 75 % when retrieve benign nodules, while when retrieve malignant nodules obtained Precision of 86 % with Recall 25 %, Precision of 74 % to the Recall 50 % and Precision of 62 % to the Recall 75 %. The PN for retrieve 30 nodules achieve Precision of 85 % for benign nodules and 87 % for malignant nodules. After update weights was achieved Precision of 95 % to the Recall 25 %, Precision of 90 % to the Recall 50 % and Precision of 70 % to the Recall 75 % to retrieve benign nodules; and Precision of 91 % to the Recall 25 %, Precision of 86 % with Recall 50 % and Precision of 67 % to the Recall 75 % to retrieve malignant nodules in the Evaluation database. In the Validation database the update weights obtained Precision 96 % to the Recall 25 %, Precision of 91 % to Recall 50 % and a Precision of 84 % to the Recall 75 % to retrieve benign nodules; and Precision of 100 % to the Recall 25 %, Precision of 92 % to the Recall 50 % and Precision of 73 % to the Recall 75 % to retrieve malignant nodules. PN achieve Precision of 95 % to retrieve 30 nodes for both malignancies.

Tables 7 and 8 present a summary of the evaluation results in the Validation database using PR and PN from InV vector with and without update weights respectively.

**Table 7:** Summary of results using PR and PN obtained with the InV vector without update weights applied to Validation database

	Recall		
	25 %	50 %	75 %
Precision to benign	82 %	66 %	52 %
Precision to malignant	86 %	74 %	62 %
	Precision (*n*=30)		
Benign	85 %		
Malignant	87 %

**Table 8:** Summary of results using PR and PN obtained with the InV vector with update weights applied to Validation database

	Recall		
	25 %	50 %	75 %
Precision to benign	95 %	90 %	70 %
Precision to malignant	91 %	86 %	67 %
	Precision (*n*=30)		
Benign	95 %		
Malignant	95 %		

Although perceived influence of parameters values used in the upgrade process, especially related to the number of iterations required to reach the best result, it is not possible to measure this influence in a scalar value, i.e. it is perceived empirically, but we could not describe it quantitatively.

The results achieved by adjusting the weights in vector 3D MSA can be compared with the results presented by [12], although the evaluation method was another. The evaluation method used was Normalized Discounted Cumulative Gain (NDCG) reaching a score of 85 % to retrieve pulmonary nodules.

The paper presented by DHARA et al. [14], which used linear regression to reduce the dimensionality of vector attributes of texture and shape, presented results that were measured by the calculation of Precision when retrieve 5 similar nodules. The average precision achieved by them in the recovery of 40 nodules from LIDC-IDRI nodules was 72.18 %.

SEITZ et al. [15] determined an ideal vector representation from a set of 63 attributes extracted from the texture, shape, size and intensity. To select the best attributes, they used genetic algorithms to find the best combination of attributes. The results were evaluated by calculating the average precision to retrieve 3, 5, 10, 20 and 50 images. The best result reaches the average precision of 86.91 % to retrieve 3 nodules through a vector composed of 29 attributes, among the 63 initials.

KURUVILLA & GUNAVATHI [16] tried to find the ideal vector for the representation of nodules in CBIR systems by calculating the accuracy in the Neural Network algorithm. They evaluated different similarity metrics to identify one that provides higher precision in retrieval of similar nodules. The results were evaluated by calculating precision. The best results achieved 95 % in average precision using the parameters indicated by the Neural Network and having the function Bray-Curtis as similarity metrics.

Analysing results, the vector that achieve best results to retrieve nodules is that formed by 3D MSA with the updated weights. It achieve Precision of 100 % to retrieve benign and malignant pulmonary nodules to the Recall 98 % for Evaluation and Validation database. The comparison between the results obtained using the initial weights and the updated weights demonstrate the effectiveness of the algorithm and its capability to produce good results.

## Conclusion

This paper presented an algorithm to automatic update weights using WED in order to improve the precision to retrieve pulmonary nodules in CBIR systems whose similarity metric was defined in the multi-dimensional vector space. As a basis for the development of such proposal, was used a generic architecture of CBIR system. For representation of nodules were used the 3D TA, 3D MSA and InV vector. The similarity metric was the DEP and to measure the weights was used ISD. Thus, we achieved better results with adjusted weights than those that have been achieved using a similar architecture but without the concept of weights associated with the attributes presented here.

Based on the results, WED applied to the three vectors used attributes (3D TA, 3D MSA and InV), with weights adjusted by the process, always achieved better results than those found with ED, i.e. without weight adjustments [36]. With the weights, the Precision was increased on average by 17.3 % compared with using ED. This improvement was observed taking into account the nodules classified in 4 malignancies (1, 2, 4 and 5) grouped into two classes being 1 and 2 classified as benign and 4 and 5 classified as malignant. This confirms the hypothesis that the WED provide better results than ED.

Finally, the analysis of the weights of the attributes was not possible to determine which attributes that are indicative of any classes (benign or malignant), i.e. it is not possible to determine that certain attribute is good or bad to determine the classification of nodules, since that in update weights method there is no correlation of the weights with the nodule class. Updating weights only standardize the sample recovered by emphasizing the attributes whose values are more similar and deemphasizing attributes whose values are widely dispersed. This emphasis does not match indication of malignancy.

### Limitation

Although Precision has achieved 100 % to retrieve up to 30 nodules, we believe that the algorithm can be improved. The result was achieved by evaluating the ranking of nodules in benign and malignant, however, nodules from LIDC-IDRI database used in training is classified according to the likelihood of malignancy that is determined in five different classes. So we believe that evaluating as a multiclass problem the results could be more precise. This can be achieved by inserting two factors that have not yet been applied to our solution: the insertion of randomness in the selection of the weights to update them through the use of search algorithms to minimize the possibility of the occurrence local maximum results; and creating a correlation between the update weights and nodule malignancy in the update process, which could improve through the insertion of this important information for the recovery method.

Moreover, the recovery of nodules in this study was restricted to the analysis of the likelihood of malignancy. There was not a visual analysis by experts to check whether the retrieved similar nodes are similar due to the similarity of vectors and visually according to the visual characteristics used by experts.

Another factor that is important is the analysis of other similarity metrics such as the Manhattan Distance, Mahalanobis Distance, Variance Weighted Average, and other metrics defined in the vector space with the application of the concept of weights presented in this work.

## Nomenclature

*CT* Computed Tomography*CAD* Computer-Aided Diagnosis*CBIR* Content-Based Image Retrieval*TA* Texture Attributes*MSA* Margin Sharpness Attributes*ED* Euclidean Distance*WED* Weighted Euclidean Distance*COM* Coocurrence Matrix*LIDC-IDRI* Lung Image Database Consortium and Image Database Resource Initiative*SVM* Support Vector Machine*RBF* kernel Radial Basis Function*AUC* Area Under Curve*KNN* K-Nearest Neighbors*WA* Weighted Average*LWR* Locally Weighted Regression*NDCG* Normalized Discounted Cumulative Gain*LWL* Locally Weighted Learning*LOO* Leave-One-Out*ISD* Inverse Standard Deviation*SD* Standard Deviation*PR* Precision vs. Recall*PN* Precision(*n*)*HUPAA* University Hospital Professor Alberto Antunes
